# The prognostic value of measurement of high-sensitive cardiac troponin T for mortality in a cohort of stable chronic obstructive pulmonary disease patients

**DOI:** 10.1186/s12890-016-0319-9

**Published:** 2016-11-25

**Authors:** Anke Neukamm, Gunnar Einvik, Arne Didrik Høiseth, Vidar Søyseth, Nils Henrik Holmedahl, Natalia Kononova, Torbjørn Omland

**Affiliations:** 1 Department of Imaging, Akershus University Hospital, Lørenskog, Norway; 2 Division of Medicine, Akershus University Hospital, Lørenskog, Norway; 3 Department of Cardiology, Oslo University Hospital, Oslo, Norway; 4 Glittreklinikken, LHL-klinikkene, Hakadal, Norway; 5 Institute of Clinical Medicine, University of Oslo, Oslo, Norway

**Keywords:** Chronic obstructive pulmonary disease, Cardiac troponin T, Mortality, Biomarker

## Abstract

**Background:**

Cardiovascular disease (CVD) is a common comorbidity in chronic obstructive pulmonary disease (COPD). Cardiac troponin (cTn) elevation, indicating myocardial injury, is frequent during acute COPD exacerbations and associated with increased mortality. The prognostic value of circulating cTnT among COPD patients in the stable state of the disease is still unknown.

The purpose of the present study was to assess the association between circulating cTnT measured by a high sensitive assay (hs-cTnT) and all-cause mortality among patients with stable COPD without overt CVD.

**Methods:**

In a prospective cohort study we included 275 patients from the Akershus University Hospital’s outpatient clinic and from Glittre, a pulmonary rehabilitation clinic. COPD-severity and cardiovascular risk factors were assessed, and time to all-cause death was recorded during a mean follow-up time of 2.8 years.

**Results:**

One hundred-eighty patients (65%) had hs-cTnT concentrations ≥ the level of detection (5.0 ng/L) and 66 patients (24%) had hs-cTnT above the normal range (≥14.0 ng/L). In total, 47 patients (17%) died. hs-cTnT concentrations in the ranges <5.0, 5.0–13.9 and ≥14 ng/L were associated with crude mortality rates of 2.8, 4.4 and 11.0 per 100 patient-years, respectively. In adjusted analyses the hazard ratios (95% confidence intervals) for death were 1.7 (0.8–3.9) and 2.9 (1.2–7.2) among patients with hs-cTnT concentrations 5.0–13.9 and ≥14 ng/L, respectively, compared to patients with hs-cTnT <5.0 ng/L.

**Conclusions:**

hs-cTnT elevation is frequently present in patients with stable COPD without overt CVD, and associated with increased mortality, independently of COPD-severity and other cardiovascular risk factors.

**Electronic supplementary material:**

The online version of this article (doi:10.1186/s12890-016-0319-9) contains supplementary material, which is available to authorized users.

## Background

Chronic obstructive pulmonary disease (COPD) is a progressive disease with increasing prevalence [1]. COPD is associated with severe comorbidities that influence prognosis, including cardiovascular disease (CVD) [2]. While smoking is a shared risk factor for both COPD and CVD, COPD has also been described as an independent risk factor for the development of CVD [3, 4] and it has repeatedly been shown that CVD is more common among COPD patients than in the general population [5–7]. Systemic inflammation, oxidative stress and hypoxemia may contribute to the development of CVD in this patient group [8].

Measurement of cardiac specific troponins (cardiac troponin T and troponin I (cTnT, cTnI)) is incorporated in the definition and diagnosis of acute myocardial infarction (MI) [9]. Other acute cardiac and non-cardiac conditions not directly related to MI have also been associated with higher troponin levels, such as acute heart failure, peri-/myocarditis, pulmonary embolism, renal failure, sepsis, and even strenuous exercise [10, 11]. In addition, chronic, low-grade elevations of cardiac troponins have been observed in patients with stable coronary artery disease and chronic heart failure [12, 13]. Previously, COPD has been associated with elevated cTnT in the exacerbated, as well as in the stable state of the disease [14–17]. Moreover, with highly sensitive methods available, it has been shown that even levels of cTnT in the low-concentration range are associated with increased mortality among patients with acute exacerbations of COPD (AECOPD) [14, 15, 18]. However, to the best of our knowledge, the association between cTnT measured during the stable state of the disease and mortality has not been studied previously. Accordingly, the objective of the present study was to relate cTnT measured in patients with stable COPD and mortality while controlling for relevant confounding factors.

## Methods

### Design and participants

This was a prospective cohort study including 275 patients with stable COPD from the outpatient clinic at the Akershus University Hospital and from Glittre Hospital, a pulmonary rehabilitation hospital in Akershus, Norway. Patients were included from June 2009 through July 2013 and January 2010 through June 2011, respectively. A stable state was defined as not having an AECOPD or being recovered from an AECOPD at least 3 weeks prior to inclusion. Patients with previously established CVD (defined as history of MI, angina pectoris, percutaneous coronary intervention or stroke) were excluded (*n* = 16) in the present analyses. Further details of the recruitment procedure have been reported previously [16, 19, 20].

### Data collection at baseline

History of smoking, hypertension, diabetes and use of antiplatelet therapy as well as function status by the modified Medical Research Council (mMRC) dyspnoea scale were obtained by interview. Systolic blood pressure, weight and height were measured, and BMI calculated. The presence of left ventricular hypertrophy (LVH, Sokolow-Lyon criteria), pathological Q- and T-waves and resting heart rate were evaluated by two physicians (AN, GE), blinded by troponin levels and outcome. The arterial partial pressures of oxygen and carbon dioxide (PaO2 and PaCO2) were obtained from radial arterial punctures and post-bronchodilatory spirometry measures, i.e. forced expiratory volume in 1 second (FEV1) and forced vital capacity (FVC) were registered from the best of 3 measures. Six-minute walking distance (6MWD) was performed. Haemoglobin and leucocytes count were analysed from antecubital venous punctures, while serum and plasma samples were stored at −80 °C pending analysis of creatinine and hs-cTnT (cobas e 411 immunoanalyzer, Roche diagnostics). According to the manufacturer of the hs-cTnT assay, the lower limit of detection is 5.0 ng/L, and the 99^th^ percentile in a sample of 533 healthy volunteers was 14 ng/L. The lowest hs-cTnT level with 10% coefficient of variation was 13 ng/L.

### Study endpoint

Mortality data was gathered from the National Population Registry, which is based on a unique personal identification number for all Norwegians. The censoring data (end of follow-up) was set to November 17, 2014.

### Ethical considerations

The study was approved by the Data Protection Authority, and reviewed by the Regional Committee for Research Ethics. All patients gave their written consent prior to participation.

### Statistical analyses

hs-cTnT levels were categorized in three groups: <5.0 ng/L, 5.0-13.9 ng/L and ≥14 ng/L. Additionally, the data was analyzed using hs-cTnT as a continuous variable, logarithmically transformed due to skewed distribution. Assuming a mortality rate at 24 months of follow-up of 0.25 in patients with troponin T level values > 0.013 ng/L (assumed *n* = 100) and 0.13 in patients with troponin level values <0.013 ng/mL (assumed *n* = 100), the study will have >80% power to detect a significant difference (alpha = 0.05).

The analyses were performed in four steps. First, univariate associations between hs-cTnT and covariates at baseline were assessed using Chi-square test, one-way analysis of variance or Kruskal-Wallis test for categorical and continuous variables, respectively. Second, age-adjusted log-rank test for mortality was performed for covariates if the association between the covariate and hs-cTnT in the univariate analysis had a p-value < 0.2. Third, if the age-adjusted log-rank test for mortality for each covariate revealed a p-value <0.2, we calculated the crude and adjusted mortality rate ratios (MRR) between each level of hs-cTnT for each corresponding covariate. The Mantel-Haenszel test was used to evaluate the statistical significance by the MRR. Moreover, we tested if the Mantel-Haenszel test revealed a p-value <0.05 for homogeneity. Fourth, if the p-value of the Mantel-Haenszel test was <0.2, the covariate was included in a multivariate proportional hazard Cox regression model subsequently reduced by backward elimination. Covariates were removed from the model if the association between a covariate and mortality were non-significant and removal of the covariate changed the estimate of coefficient between hs-cTnT and mortality < 20%.

In the first step, the univariate associations between hs-cTnT and these covariates were assessed: Gender, age, FEV_1_/FVC-ratio, FEV_1_, BMI, LVH, pathological Q and T-wave on the electrocardiogram, history of hypertension, history of diabetes, use of antiplatelet therapy, smoking status, number of tobacco pack years, heart rate, systolic BP, haemoglobin, leucocytes, PaO2_,_ PaCO2 and 6MWD. Glomerular filtration rate (GFR) was estimated by the Cockcroft-Gault equation [21]. Before the subsequent steps, continuous covariates were categorized. The limits were guided by clinical practice and distribution in the sample: Age was categorized in three groups: <62 years, 62–66 years and ≥67 years. Spirometry measures were categorized by GOLD class, heart rate in <71/min and ≥71/min (median value), systolic blood pressure in <120 and ≥120 mmHg (median value), leucocyte count in <7×10 and ≥7×10^9^/L (median value), GFR in <45, <60 and ≥60 mL/min, partial pressure of oxygen (PaO2) in <8 kPa and ≥8 kPa and 6 minute walking distance was categorized in <450 and ≥450 m (median value), respectively. The analyses were performed using STATA 13.1 (Stata Corp LP, Texas, USA) and SPSS (Version 20; IBM Corp, New York, NY, USA).

## Results

The median hs-cTnT was 6.3 ng/L (interquartile range 5–13 ng/L). The prevalences of patients having hs-cTnT <5.0 ng/L, 5.0–13.9 ng/L and ≥14 ng/L were 35%, 41% and 24%, respectively. Mean age (SD) at inclusion was 64 (7.2) years and 146 (53%) were female. Univariate associations between hs-cTnT categories and covariates using the inclusion criterion of a *p*-value <0.2 were observed for the following covariates (Table 1): Age, gender, GOLD class, history of hypertension and diabetes, current smoking, systolic blood pressure, heart rate, pathological Q-wave, PaO2, leucocyte count, GFR, 6MWD and mMRC-score.

**Table 1:** Distribution of TnT by relevant covariates at baseline

Covariate	High sensitivity Troponin T, ng/L			*p*-value*
	<5.0 (*n* = 95)	5.0–13.9 (*n* = 114)	≥14.0 (*n* = 66)	
Age, years, mean (SD)	62 (7.3)	64 (6.7)	67 (7.2)	<0.001
Female, n (%)	64 (67)	56 (49)	26 (39)	<0.001
BMI, mean (SD)	24.7 (5.1)	24.7 (5.4)	24.5 (5.4)	0.962
GOLD class III/IV, n (%)	46 (48)	60 (53)	51 (77)	0.001
Hypertension, n (%)	32 (34)	36 (32)	36 (55)	0.006
Diabetes, n (%)	4 (4)	1 (1)	9 (14)	0.001
Current smoking, n (%)	34 (36)	29 (26)	11 (17)	0.025
Pack years, mean (SD)	35 (16.1)	35 (16.9)	37 (19.5)	0.686
Systolic BP, mmHg mean (SD)	132 (21.9)	138 (20.1)	138 (19.5)	0.080
Hemoglobin, g/dL mean (SD)	14.1 (1.2)	14.3 (1.1)	15.7 (13.7)	0.285
Heart rate per min, mean (SD)	70 (13.3)	71 (12.9)	75 (13.9)	0.067
LVH, n (%)	1 (1)	5 (5)	2 (3)	0.358
Pathologic Q-wave, n (%)	5 (5)	6 (5)	9 (14)	0.067
Use of antiplatelet therapy, n (%)	10 (10)	12 (11)	5 (8)	0.781
PaO2, kPa, mean (SD)	9.0 (1.1)	9.3 (1.3)	8.9 (1.3)	0.058
PaCO2, kPa, mean (SD)	5.4 (0.7)	5.4 (0.8)	5.5 (0.8)	0.847
Leucocytes, x10^9^/L, mean (SD)	7.0 (1.7)	7.1 (1.9)	7.8 (2.3)	0.028
GFR, mL/min, mean (SD)	89 (3)	89 (3)	79 (4)	0.044
6MWD, meters, mean (SD)	465 (109)	455 (123)	365 (104)	<0.001
mMRC >2, n (%)	42 (44)	58 (51)	47 (71)	0.003
AECOPD during last year, n (%)	33 (35)	40 (36)	28 (43)	0.571

*Analysis of variance, Pearson Chi-Square, Fisher’s exact test, as appropriate

The median time to death or censoring time was 2.8 years. 47 patients (17.1%) died during the follow-up period. The crude mortality rates per 100 patient-years (95% confidence interval) were 2.8 (1.4–5.3), 4.4 (2.7–7.0) and 11.0 (7.2–16.9) in patients with hs-cTnT <5.0 ng/L, 5.0–13.9 ng/L and ≥14 ng/L, respectively.

**Table 2:** Mortality rates (MR) by relevant categorical covariates

Covariate	m (MR)	RR	*p*-value*	
			unadjusted	Age-adjusted
Age, years		1.81	0.002	-
< 62	8 (2.6)			
62–66	12 (4.2)			
≥ 67	27 (8.7)			
Gender		1.30	0.416	0.379
Male	19 (4.5)			
Female	28 (5.8)			
Current smoking		0.93	0.864	0.768
Yes	11 (4.9)			
No	36 (5.3)			
Hypertension		1.14	0.658	0.936
Yes	19 (5.6)			
No	28 (5.0)			
Diabetes		2.94	0.007	0.011
Yes	6 (14.1)			
No	41 (4.8)			
GOLD class		2.95	0.002	0.004
I–II	10 (2.5)			
III–IV	37 (7.4)			
PaO2 < 8 kPa		1.65	0.134	0.336
Yes	12 (7.7)			
No	35 (4.7)			
Pathologic Q-wave		2.39	0.028	0.071
Yes	7 (11.6)			
No	40 (4.9)			
Antiplatelet therapy		0.81	0.690	0.791
Yes	4 (4.3)			
No	43 (5.3)			
Leucocytes ≥ 7 × 10^9^/L		2.52	0.004	0.003
Yes	34 (7.4)			
No	13 (2.9)			
Heart rate >71 per min		2.48	0.004	0.004
Yes	34 (7.3)			
No	13 (2.9)			
Systolic blood pressure >140 mmHg		1.33	0.583	0.999
No	6 (4.1)			
Yes	41 (5.4)			
GFR		0.40	0.008	0.025
< 45 mL/min	4 (24.5)			
45–60 mL/min	8 (6.4)			
> 60 mL/min	35 (4.6)			
6MWD < 450 m		3.63	<0.001	0.002
Yes	35 (8.7)			
No	12 (2.4)			
mMRC > 2		2.17	0.015	0.012
Yes	33 (7.0)			
No	14 (3.2)			

*Log-rank test

Age-adjusted associations between covariates and mortality were observed for history of diabetes, GOLD-class, pathological Q-wave, leucocyte count, creatinine, heart rate, 6MWD and mMRC >2 (Table 2)﻿. The number of deaths and mortality rates across hs-cTnT levels, stratified by the covariates significantly associated with hs-cTnT, are shown in Table 3.

**Table 3:** Mortality, mortality rates, mortality rate ratio expressed as score test for trend for a one unit increase in troponin category, by selected covariates

	High-sensitivity troponin, ng/L			MRR (95% CI)	
	<5.0	5.0–13.99	≥14		
	(*n* = 95)	(*n* = 114)	(*n* = 66)		
Covariate	m (MR)	m (MR)	m (MR)	Unadjusted	Adjusted^a^
All, crude	9 (2.8)	17 (4.4)	21 (11.0)	2.1 (1.4–3.1)	-
Age at inclusion, years					
< 62	2 (1.4)	3 (2.4)	3 (7.0)	2.4 (0.9–6.6)	1.9 (1.3–2.8)
62–66	3 (3.1)	4 (3.2)	5 (7.8)	1.7 (0.8–3.6)	
≥ 67	4 (4.6)	10 (7.2)	13 (15.5)	1.9 (1.1–3.1)	
Gender					
Female	8 (3.7)	9 (4.7)	11 (15.3)	2.2 (1.3–3.8)	2.3 (1.5–3.4)
Male	1 (0.9)	8 (4.1)	10 (8.4)	2.3 (1.2–4.3)	
Diabetes					
Yes	0 (0)	0 (0)	6 (25.7)	2.5 (1.1–5.9)	1.9 (1.3–2.8)
No	9 (2.9)	17 (4.5)	15 (8.9)	1.8 (1.2–2.7)	
GOLD class					
I–II	2 (1.1)	4 (2.2)	4 (9.5)	3.4 (1.3–8.7)	1.8 (1.2–2.6)
III–IV	7 (4.6)	13 (6.4)	17 (11.4)	1.6 (1.1–2.4)	
PaO2 < 8 kPa					
Yes	3 (5.3)	2 (3.2)	7 (19.6)	2.3 (1.1–4.8)	2.1 (1.4–3.0)
No	6 (2.2)	15 (4.6)	14 (9.0)	2.1 (1.4–3.1)	
Pathologic Q-wave					
Yes	1 (7.4)	1 (5.2)	5 (17.9)	1.7 (0.7–4.4)	2.0 (1.4–2.9)
No	8 (2.6)	16 (4.4)	16 (10.3)	2.1 (1.4–3.2)	
Leucocytes > 7×10^9^/L					
Yes	6 (3.6)	12 (6.7)	16 (14.2)	2.0 (1.3–3.1)	2.0 (1.4–2.9)
No	3 (1.9)	5 (2.4)	5 (6.4)	1.9 (0.9–4.2)	
Heart rate, > 71 per min					
Yes	7 (4.8)	11 (5.9)	16 (12.2)	1.7 (1.1–2.6)	1.9 (1.3–2.8)
No	2 (1.1)	6 (3.0)	5 (8.3)	2.9 (1.3–6.4)	
GFR					
< 45 mL/min	0 (0)	1 (12.1)	3 (37.2)	2.8 (0.4–19.8)	1.9 (1.2–3.0)
45–60 mL/min	1 (1.7)	3 (9.3)	4 (11.4)	2.2 (1.0–5.0)	
> 60 mL/min	8 (3.0)	9 (3.7)	14 (9.5)	1.9 (1.2–2.9)	
6MWD, <450 m					
Yes	7 (5.6)	12 (7.8)	16 (12.6)	1.5 (1.0–2.3)	1.7 (1.2–2.5)
No	2 (1.0)	5 (2.1)	5 (7.8)	3.1 (1.4–7.3)	
mMRC > 2					
Yes	6 (4.4)	10 (5.0)	17 (12.6)	1.8 (1.2–2.8)	1.9 (1.3–2.8)
No	3 (1.6)	7 (3.7)	4 (7.1)	2.2 (1.0–4.8)	

^a^Mantel Haenzel test

Overall, the crude MRR across categories of hs-cTnT was 2.1 (95% CI: 1.4–3.1). The bivariate analyses show that the crude and adjusted MRR were similar, thus, there was no meaningful confounding in the data regarding mortality. The Mantel-Haenszel test did not reveal a *p*-value <0.05 for homogeneity for any of the covariates which is why we assume that there was no effect modification of relevant variables.

Age, gender, GOLD class, 6MWD, history of diabetes, leucocyte count and pathological Q-wave were included in the final Cox regression model (Table 4).

**Table 4:** Multivariate adjusted hazard ratios for all-cause death

Variable	HR^a^	95% CI^a^
Hs-cTnT, ng/L		
< 5	1	
5–13.9	1.7	0.8–3.9
≥ 14	2.9	1.2–7.2
p for trend	<0.001	
Age		
< 62	1	
62–66	1.6	0.6–3.9
≥ 67	2.7	1.2–6.0
p for trend	0.002	
Female versus male^b^	1.9	0.99–3.8
Gold class III/IV versus I/II	1.8	0.84–3.8
Pathologic Q-wave, yes versus no	1.5	0.62–3.6
Diabetes, yes versus no	1.9	0.8–5.1
Leucocyte count > 7×10^9^/L, yes versus no	2.3	1.2–4.6
6MWD < 450 m, yes versus no	1.6	0.7–3.3

^a^Cox regression analysis

^b^violated the proportional hazard assumption, i.e. the HR changed during the follow-up

There was a positive association between hs-cTnT and mortality (p for trend < 0.001). In adjusted analyses, using patients with hs-cTnT <5.0 ng/l as the reference, the hazard ratios (95% confidence intervals) for death were 1.7 (0.8–3.9) and 2.9 (1.2–7.2) among patients with hs-cTnT concentrations 5.0–13.9 and ≥14 ng/L, respectively. We also investigated the association between mortality and hs-cTnT expressed as a continuous variable. One unit increase in the natural logarithm of hs-cTnT in the final Cox model was associated with a hazard ratio (95% CI) of 1.7 (1.2–2.4). We observed no significant changes in the results after exclusion of two patients with hs-cTnT values >100 ng/L. The global test of the model did not violate the proportional hazards assumption, neither did the single covariates with the exception of gender. However, gender did not influence the association between troponin and mortality.

## Discussion

The main finding in this study is that cTnT-elevation above the 99^th^ percentile in patients with stable COPD without overt CVD is associated with increased mortality, independently of COPD-severity and traditional cardiovascular risk factors.

While the diagnostic properties of elevated troponins are important in patients presenting with chest pain, the properties as a prognostic marker have received considerable interest in acute coronary syndrome, as well as in other conditions such as stable coronary heart disease, heart failure, pulmonary embolism, renal failure, ischemic stroke and sepsis [12, 22–27]. To the best of our knowledge this is the first prospective study to assess the association between cTnT and long-term mortality in patients with stable COPD. The present finding is in line with studies in other patient populations in which small increments of circulating concentrations of cTnT are associated with poor outcome above and beyond traditional risk factors [12, 28, 29]. The fact that hs-cTnT in our population was associated with total mortality independently of strong predictors of mortality such as the components of the BODE index, makes hs-cTnT a possible candidate for early risk prediction in stable COPD patients.

Given that COPD is primarily a pulmonary disease, it is of interest to consider the possible pathophysiological mechanisms underlying cTnT elevation in COPD. cTnT is exclusively synthesized in cardiomyocytes, and the presence of cTnT in serum implicates leakage of cTnT into the circulation by cell damage or increased cell turnover. The most common causes for chronic, low-grade cTnT-increase are ischemic heart disease and conditions characterized by increased cardiac strain [30]. The participants in the present study were without overt CVD, but we cannot exclude the possibility that patients with increases in cTnT had undiagnosed coronary artery disease. Indeed 20 (7%) of the patients had a pathological Q-wave on the ECG, but interestingly this was not significantly related to hs-cTnT concentrations. N-terminal prohormone of B-type natriuretic peptide (NT-proBNP), an indicator of heart failure, was not measured in all participants, but a previous analysis in a subgroup (*n* = 101) showed normal NT-proBNP levels and no association with cTnT [16].

Pertinent to the hypothesis that COPD might be a risk factor for CVD, factors known to be associated with secondary cardiovascular effects, such as systemic hypoxemia and systemic inflammation, were evaluated. In the present study only 45 (16%) of the patients had arterial hypoxemia, and we found no statistically significant association between partial pressure of oxygen and troponin or mortality. Leucocyte count as an indicator of elevated baseline inflammation was associated with increased levels of troponin and risk of death in the present study. Such associations have been reported earlier [16, 31]. Inflammatory activity might promote the development and worsening of subclinical atherosclerotic disease and thereby prognosis in this population. There is also a possibility that cTnT elevation is mediated by an inflammatory effect on the cardiomyocytes. In that case, leukocyte count represents a mediator and should not been included as a covariate [32]. A higher inflammatory state has also been linked to worse prognosis and more frequent exacerbations in COPD [33].

Another possible mechanism underlying cTnT elevation in stable COPD might be increased right ventricular strain associated with pulmonary hypertension [34]. Hattori et al. have described an association between hs-cTnT with right heart pressure in stable COPD [31]. It is conceivable that the involvement of the right heart accounts for both elevated hs-cTnT and increased mortality in stable COPD independently of lung function.

The current study extends previous data concerning the prognostic value of hs-cTnT in patients with acute exacerbation of COPD. However, although elevated cTn concentrations are associated with poor outcome, it is not established how clinicians should approach this situation neither in COPD nor in other patient groups.

### Study limitations

In order to further evaluate the discriminative power of cTnT in risk stratification, larger study samples are needed. Serial hs-cTnT measurement would provide us with a better possibility to evaluate the associations between cTnT and mortality in relation to COPD-progression and other cardiovascular risk factors. The study would have benefitted from baseline cardiac imaging, both with regard to undiagnosed coronary heart disease and myocardial function. A stable state was defined as <3 weeks since last exacerbation. This is a shorter period than in several other COPD-studies. However, excluding patients having an exacerbation during the last 3 months prior to inclusion did not change the main results (data not shown). We lack information regarding the cause of death, which could have given us additional insight in the relation between cTnT and cardiovascular deaths.

## Conclusion

cTnT, measured by a high sensitivity assay, was associated with increased all-cause mortality, independently of COPD-severity.

## Additional file

Additional file 1: Data set. (XLS 110 kb)

## Abbreviations

6MWD: 6-minutes walk-test
BMI: Body mass index
CI: Confidence intervals
COPD: Chronic obstructive pulmonary disease
CVD: Cardiovascular disease
ECG: Electrocardiography
FEV1: Forced expiratory volume during 1^st^ second
FVC: Forced vital capacity
GFR: Glomerular filtration rate
GOLD: Global initiative of obstructive lung disease
HR: Hazard ratio
hs: High-sensitivity
LVH: Left ventricular hypertrophy
MI: Myocardial infarction
mMRC: Modified Medical Research Council
MRR: Mortality rate ratio
NT-proBNP: N-terminal pro B-type natriuretic peptide
SD: Standard deviation
TnT/TnI: Troponin T/I
